# Designing Mobile Applications for Emergency Response: Citizens Acting as Human Sensors

**DOI:** 10.3390/s16030406

**Published:** 2016-03-19

**Authors:** Marco Romano, Teresa Onorati, Ignacio Aedo, Paloma Diaz

**Affiliations:** Computer Science Department, Universidad Carlos III de Madrid, Avda. de la Universidad 30, 28911 Leganés, Madrid, Spain; tonorati@inf.uc3m.es (T.O.); aedo@ia.uc3m.es (I.A.); pdp@inf.uc3m.es (P.D.)

**Keywords:** emergency notification systems, mobile devices, human sensors, social software

## Abstract

When an emergency occurs, citizens can be a helpful support for the operation centers involved in the response activities. As witnesses to a crisis, they initially can share updated and detailed information about what is going on. Moreover, thanks to the current technological evolution people are able to quickly and easily gather rich information and transmit it through different communication channels. Indeed, modern mobile devices embed several sensors such as GPS receivers, Wi-Fi, accelerometers or cameras that can transform users into well-equipped *human sensors*. For these reasons, emergency organizations and small and medium enterprises have demonstrated a growing interest in developing smart applications for reporting any exceptional circumstances. In this paper, we present a practical study about this kind of applications for identifying both limitations and common features. Based on a study of relevant existent contributions in this area and our personal direct experience in developing and evaluating emergency management solutions, our aim is to propose several findings about how to design effective and efficient mobile emergency notification applications. For this purpose we have exploited the basic sensors of modern mobile devices and the users’ aptitude for using them. The evaluation consists of a practical and a theoretical part. In the practical part, we have simulated a traffic accident as closely as possible to a real scenario, with a victim lying on the ground near a car in the middle of a street. For the theoretical part, we have interviewed some emergency experts for collecting their opinions about the utility of the proposed solution. Results from this evaluation phase confirm the positive impact that EN application have for both operators’ and citizens’ perspective. Moreover, we collected several findings useful for future design challenges in the same area, as shown in the final redesign of the proposed application.

## 1. Introduction

In the last decade European Union and national governments have allocated funds to promote research in the Emergency Management (EM) area. EM is a broad research field that can significantly improve citizens’ quality of life, not only helping them during a crisis, but also through an adequate monitoring activity to prevent damages and victims [1]. One of the most critical aspects of EM is the Emergency Notification (EN) system concerning how to get updated and accurate information from the very first stages of the event and how to notify affected people. In order to support this activity, EN systems have been developed aiming at improving the performance and the efficiency of such activities. As Yuan *et al.* explain in [2], a possible approach to achieve this is by counting on the collaboration of citizens that are directly involved into the crisis acting as human sensors. In this way, it is possible to take advantage of the so called citizen journalism referring to the common practice of sharing different kinds of messages from anywhere and at any moment of the everyday life. Moreover, citizens possess the power conferred by the advances in mobile technologies. Indeed, modern mobile devices possess several built-in sensors such as cameras and microphones or others capable of measuring motion, orientation and various environmental conditions. Considering the spread of such technologies, data collected from these sensors are useful for figuring out what is happening in a specific context.

Real examples of the human sensors’ participation are given by the earthquake and subsequent tsunami that happened in 2011 in Japan. In [3], the authors discuss how people used Twitter or Skype to share positions, texts and photos and to stay in contact with their families. One of the most interesting findings of Ichiguchi was the impressive amount of information shared on Twitter that forced the Japanese government to open a specific account for this event and to promote the usage of predefined hashtags. Consequently, to avoid connectivity problems the Wi-Fi hotspots of fast-food restaurants, hotels, public infrastructures and even private flats were temporarily opened up. In this way, citizens had the possibility to collect a great amount of information, including not only text messages but also photos, videos and GPS positions.

People’s flair for using pervasive mobile technologies to share important information encourages researchers to find solutions based on them. Gathered information can be used for both long-term or short-term solutions. In the first case, the information is used to follow the evolution of a specific crisis and eventually to learn from it for future emergencies. An example is represented by the web application eStoryS [4] that creates and visualizes storyboards about an emergency with the goal to inform operators to manage future events. Short-term solutions exploit data coming from citizens to improve the current emergency response. The most common approach in this case is to establish a bidirectional channel between EM operators and citizens for receiving and sending information in real time through an Emergency Communication Systems [5]. Other solutions are based on a one-way channel that transmits emergency messages to a specific receiver or a group of people. Such systems are called Emergency Notification Systems [6] and they are the ones most widely used nowadays thanks to the diffusion of mobile technologies. As a demonstration of their spread, in the mobile market it is already possible to find a large number of such applications. In this paper, we focus on the short-term approach and in particular on applications used by citizens for notifying emergency organizations about an exceptional situation.

Another characteristic to take into account solutions based on the information collected by citizens is the scale of the event. The contributions in literature mostly consider large-scale crises, as for example the Japan tsunami in 2011. This kind of events generates a great quantity of information, reaching the order of millions of messages shared on the most popular social networks, as shown in the analysis performed by the Pew Research Center about the usage of Twitter during Hurricane Sandy in 2012 [7]. This information is not easy to exploit in small-scale events where the number of the messages is small compared to the volume of the social network content. Therefore, literature about small-scale events is generally poor even if such events are essentially the most common that emergency organizations have to face daily. The EN applications came into the world specially to fill this gap.

The objective of this work is to analyze existing EN applications to understand their real utility, potential benefits and limitations. In literature it is possible to find examples of software developed for supporting the notification mechanism during critical situations, including both mobile clients for social networks and native applications. Main results from their analysis concern the relevance of retrieving the event and user coordinates, and using multimedia. On the other side, a poor design of the interfaces that in particular makes the interaction slow and less intuitive is noticeable**.** Our research question stands in understanding how to design mobile applications for an efficient and effective notification of emergency information from both the citizens’ and the emergency operators’ point of view.

The research process adopted for proposing a solution to this problem is based on the Design Science Research (DSR) methodology by Hevner and Chatterjee [8]. DSR is a useful approach that allows researchers to deal with real-world problems by a pragmatic research paradigm that involves the development of innovative Information Technology artifacts. Basically, the idea underlying this approach is that “you build an artifact to learn from it” by Romano [9]. In other words, researchers follow this methodology to better clarify what is new in their design.

Based on the DSR approach, first of all we perform a comprehensive review and analysis of the problem domain identifying the common characteristics (e.g., the possibility to send geo-tagged photos to the EM authorities), limitations (e.g., the complexity of the interaction) and other interesting issues of existent social software for EN. On the basis of this analysis we design a new application for the emergency notification. The proposed solution will be evaluated both quantitatively and qualitatively in order to finally understand which features are expected by emergency organizations, their usefulness in the real world and the level of acceptance of common citizens.

Thanks to the literature analysis, the experience gained with the proposed design, the results obtained from the evaluation phase and the expert interviews, we point out also several findings tailored to support developers in enhancing the design of more effective mobile EN applications. Our targets are especially software practitioners that do not have enough resources in terms of knowledge and experience to overcome weak spots commonly found during the development of such solutions.

In Section 2 we present and analyze the main characteristics of the mobile applications used to send EN. In Section 3, we describe the design of our application that is built on the basis of the common features of the main EN applications. Section 4 and Section 5 describe the study of the application and discuss the results. In Section 6 we present the redesign of the application based on the previous analysis. Finally, in Section 7 some remarks and conclusions are given.

## 2. Software for Emergency Notification

The participation of citizens in the response activities for managing a critical situation could represent a helpful support for emergency operators. Citizens could be directly involved both as victims or witnesses and thus be aware of updated information useful for decision making purposes. As stated by Sutton [10], tweets shared during an emergency come mainly from witnesses that are not directly affected by the situation, but the content is still considered crucial. For this reason, emergency agencies and organizations have been proposing the development of social software for collecting emergency notifications sent by citizens.

Social software describes web-based applications that support the exchange of user-generated content, the management of relationships and the communication in social contexts [11,12]. Nowadays, their usage is almost universal and as a consequence they have been used for collecting different kinds of information, as political preferences, opinions about some interesting event or marketing preferences. In the emergency field, social applications have been already used for more than 10 years as means to analyze and even deal with big crisis situations. Such trend is well documented by the international literature. In 2012, Reuter *et al.* [13] presented an interesting and comprehensive survey on the usage of social software during emergencies. One of their results concerns the great value that people have as source of information since during a crisis they spontaneously publish photos, videos, positions and comments [13]. A significant example is given by the citizens’ reaction to the terrorist attack of 11 September 2001. Wikis were spontaneously created to allow to collect information on missing people [14]. Another interesting example is Twitter Earthquake Detector (TED) developed by Guy *et al.* [15]. This platform allows the user to store and analyze tweets about earthquakes and comparing them with official scientific reports. This is clearly useful during the first phases of the emergency when tweets are disseminated while official data are still poor.

Other authors have been working on this topic, focusing their contributions in understanding the role of social networks and the quality of shared information. Mills *et al.* [16] pointed out that when an emergency occurs, during the first hours the content quality published in Twitter is higher than the mainstream media. Actually, Twitter replaces traditional media when they are not satisfactory for knowing more about a situation [10]. The other issue about the information coming from social networks is the reliability. Several authors are working on systems that rely on the definition of a collective intelligence that prevents false information to be spread, as stated in [17,18]. In [19] the authors state that it is necessary to discern the content related to a specific emergency and discard the remaining through the identification of relevant keywords or promoting the usage of standard hashtags as reported in [20]. Moreover, to the best of our knowledge the most interesting results have been obtained analyzing the usage of social networks for large-scale crisis, such as terrorist attacks, earthquakes or tsunamis. Data about small-scale emergencies, like a common traffic accident, normally cannot be captured because the number of related tweets is not significant.

For these reasons, practitioners are moving towards the development of dedicated mobile applications, trying to keep the characteristics of the social software but with the goal to be also useful for small-scale events. Among mobile EN applications already available in the world mobile market, we selected the most relevant ones for our scope, getting a list of eight elements (as shown in Table 1). 

In particular, we based our choice on three main issues: sending short-term notifications of small-scale events, developed and promoted by official emergency organizations or big enterprises, and used in real use cases for enhancing the citizen participation. Our aim is to identify their main characteristics and limitations that could be relevant for this work. The analysis focuses mainly on bringing to light the kind of interaction, the functionalities, the content that can be shared and the recipient of the message.

The first considered application is SafetyGPS (developed by Eugenio Herrero.; this project is currently finished and the application is no longer available), a Twitter based platform developed in Spain for sending tweets to a list of local official accounts of some city halls. In Spain, there are already several local governments that are using it for receiving real emergency notifications with a good acceptance by citizens. The application is based on an interface console in which users have to write a notification using a specific hashtag code to contact with the right authority. This means that users have to know how the hashtag code works and the account of the city hall to contact. About the content of the notification, SafetyGPS also allows users to post georeferenced videos and photos.

Similar to SafetyGPS, HelpBridge (developed by Microsoft [21]) is based on the usage of social networks for sending notifications to a list of favorite accounts. Nevertheless, in this case accounts are not authorities but friends and family and the data collection is not based on a console but on a friendlier window interface. Another constraint of this application is that it is available just in the US territory.

The MotorolaAlert (developed by Motorola [22]) and EmergencyAlert (developed by the Government of the Province of Alberta in Canada [23]) applications are also limited to notifying just friends and family with a current GPS position. The main difference between them is about the user interface. The former adopts an interface with a few options and buttons associated with representative icons. The latter consists of a textual menu with several options that can make users confused.

In case of the FEMA App (developed by FEMA [24]) it consists of a shared map where both emergency operators and common citizens can add notifications and access to published ones. Apart from the map, this app also provides other functionalities like a collection of safety tips and an emergency kit with useful information about how to behave during a critical situation. If the users want to alert about an exceptional event, this amount of features could make the task difficult to complete.

Another interesting application is SignAlert (developed by the company SIGNALERT [25]) that establishes a social platform among citizens in the same neighborhood with the aim of sharing messages about any kind of disasters with photos, texts and locations. In this case, the notification does not reach any official emergency center. Moreover, users have to fill a large form with a lot of specific questions that require a previous knowledge in the emergency field.

ELERTS (developed by the ELERTS company [26]) and My112 developed by the local government of Madrid [27]) applications allow users to contact directly with an official emergency center, but they are limited to predefined locations. The ELERTS app is valid just for hospitals or colleges to notify the security department about a problem. Consequently, it can be used just for small-scale events that do not require an immediate and urgent solution. In case of My112, it establishes a bidirectional communication channel with the emergency operation center, but the notification mechanism consists of making a traditional emergency call with the additional information of the geo-localization. Similar to My112 application, several 112 Operation Centers across Europe as well as other emergency numbers have been developing their own applications as alternatives for improving the traditional emergency call. All of them offer a similar service as making calls enriched by geo-localization. In particular, we had the possibility to test in real situations the one developed by the local government of Madrid for the Spanish 112 Operation Center and called My112. For this reason, we decided to include it among the analyzed applications.

The most interesting characteristics that emerge from our analysis are related to two main aspects: the content and the interaction.

*Content.* All the applications share as common characteristic the possibility to automatically retrieve the user position thanks to the embedded GPS. Then, with the exception of those applications based on a call communication, the others allow to collect data through forms to be filled out, simple text, photos, videos or audio. Generally, more emphasis is given to forms and text rather than more advanced multimedia content.

*Interaction.* About the interaction, we notice that designers do not focus particularly on the usability. Some applications are indeed based on console interfaces that are more suitable for power users. Others are based on forms divided into several steps making the notification mechanism slow and less intuitive. In this way, they are not considering a general rule of mobile design stating that the applications must be intuitive since the first usage because mobile users are distracted by the surrounding environment [28]. This idea acquires even more relevance in crisis situations where other factors as fear or panic come into play.

## 3. Prototyping a New Emergency Notification Application

The design of our application aims at integrating the characteristics extracted from the literature analysis and overcoming identified limitations. Moreover, we have taken advantage from the collaboration with three other practitioners, two IT companies with extensive experience in developing emergency systems and the Police Department of Valencia in Spain. The involvement of domain experts, like emergency responders, firemen or medical services, is crucial for translating their experience in the design rationale.

The application was presented for the first time in [29]. It collects multimedia content, textual descriptions and users’ position. The position is retrieved automatically with the GPS or manually choosing a point in the map.

Secondly, we decide to avoid complex interactions, as console interfaces, hashtags and the selection of the final recipient. An interface console is generally complicated in particular during critical situations. Moreover, when an emergency occurs the citizen does not have to look for the appropriate hashtag or decide who the right recipient is, but just describe the emergency and specify its nature. Depending on this information, the system is in charge of addressing the notification to the right organization making the interaction easiest. For example, a first aid request will be redirected to the medical service.

Finally, we avoid also large forms that could mean a waste of time, trying to extract useful information from the phone itself or asking some questions to the user during the first time configuration. The georeferenced message composed with the application is then delivered to the operation center, where used information system for managing emergency situations will be in charge of analyzing collected data and eventually making decisions about actions to undertake.

**Figure 1:**
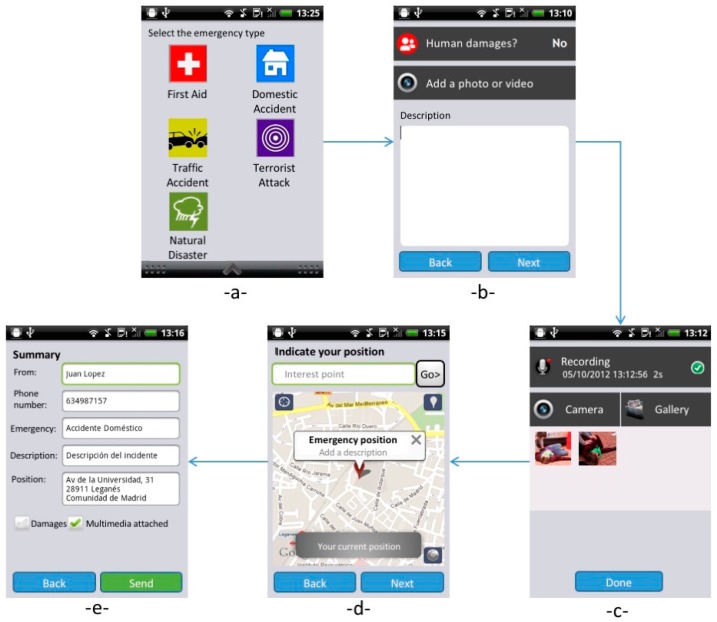
Application screen flow: -**a**- the emergency category list, -**b**- the textual description, -**c**- the multimedia content, -**d**- the localization map, -**e**- the message resume

In Figure 1, we show the flow chart of the application. In the first screen (upper left, -a- in Figure 1), users are required to choose the emergency. The different kinds of emergency were extracted from the official list used currently by EM operators of the Police Department of Valencia to categorize an event. The icons are inspired by an artwork prepared for the city of San Francisco (USA) [30] with the aim to represent different kinds of emergencies through a visual language. Moreover, we have applied the Itten’s Colour Theory [31] for a high contrast that makes the icons more recognizable by users. The Colour Theory is largely applied in the user interface design for choosing appropriate color combinations, as described in [32].

In the second view (upper right, -b- in Figure 1), users can specify if there are victims and add a textual or a multimedia description. In the following (lower right, -c- in Figure 1), the application allows to add audio recordings, pictures and videos. The fourth view (lower center, -d- in Figure 1) presents a map where the current position is shown, but the user can easily modify it if the event is located elsewhere. In this case, the notification is sent with both the current user position and the event position. The last view (-e- in Figure 1) summarizes the content of the notification to be checked by the user and finally sent. A pop-up notification will alert users when the message has been successfully processed by the EM system.

## 4. The Evaluation Study

In this section we present a user study aimed at analyzing different aspects of the mobile application: (1) the usability of the mobile interface; (2) the level of acceptance of the application by end users; (3) the real usefulness of the system; (4) the users’ behaviors while interacting with the application during an emergency.

The study is composed by two different experiments. The first one is performed in a controlled environment and it is aimed at identifying possible usability issues. The second study is carried out in a simulated environment and it is aimed at analyzing users’ behavior in a semi-controlled situation. Results obtained from both experiments have been compared in order to elicit any differences and then evaluated by experts to measure quality of gathered information and usefulness of the application and the gathered information.

### 4.1. Usability Test

In this section we present the usability study carried out on the proposed application. The goal of the study was to evaluate the suitability of the interface in terms of ease to use and user satisfaction.

We have involved workers and students from the University Carlos III of Madrid (Spain). In particular, there were ten IT users highly familiar with advanced mobile phones in order to avoid during the evaluation any problem connected to the usage of mobile technologies, such as touch screen input methods.

To evaluate the potential effectiveness and usefulness of the application, we applied a "think aloud" technique [33]. Following this approach, we asked users to perform a set of specific tasks with the current application and express their opinions out loud. In this way, as evaluators we had the possibility to collect useful suggestions about how to improve the user acceptance of the application. During the study, we focused on two main aspects of the interface:

1. The capability to understand the interface: we checked whether the layout is effective to guide users in accomplishing their tasks. The interface must not confuse users or make them feel awkward.
2. The capability to navigate the application: we needed to determine the best way to structure the navigation in order to avoid users getting lost during the interaction.

#### **Task** 

We decided to start the experiment with no preliminary training on the application, overall aiming at verifying the learnability degree of the interface. During the first part of the study, users received a short presentation about how the notification mechanism works. Then, they were introduced to an emergency scenario, a car accident with a victim, and required to act in this scenario with our mobile application. The task was divided into four subtasks (identified as 1.1, 1.2, 1.3 and 1.4 in Table 2) for a better comprehension of the different actions.

In Table 2, mean times and mean errors (the columns *untrained mean time* and *untrained mean errors*) for each subtask are compared with times and errors planned with a user who received a training session (columns *trained time* and *trained errors*). It is notable that the mean values are pretty close to the planned values. This demonstrates the ease to use and the effectiveness of the application in a normal controlled environment. Moreover, we have collected positive opinions, like “I feel really comfortable in using this application” and “I think that is intuitive, I don’t even need to learn how to use it”.

Immediately after, the participants were invited to answer to a questionnaire aimed at rating different aspects of the application using multi-choice questions with regards to reliability and easiness to understand. The multi-choice questions allowed to guide the users through different responses looking for the most representative one for their particular experience [33].

**Figure 2:**
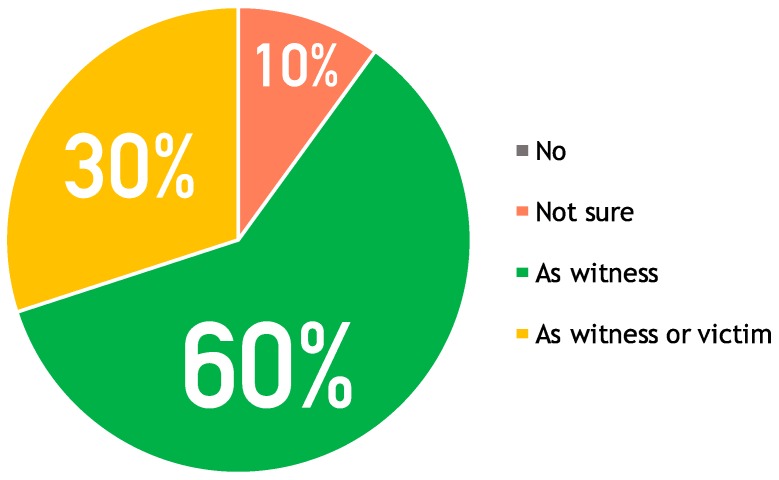
Overall user acceptance (would you use it?) of the proposed mobile application from the usability test.

The questionnaire had two parts: general opinion about the app and specific opinion for each screen in the app. In the first part, users answered four questions about the general characteristics of the application, including easiness and consistency in performing a task, trustability and acceptance. Analyzing the answers, the last question is the most representative one for understanding the general users’ opinion, as shown in the pie chart in Figure 2. The pie chart has four areas: *no* for “I’m not going to use it” has a 0% rate (grey), *not sure* for “I’m not sure” has a 10% rate (coral), *as witness* for “I would use it as witness” has a 60% rate (green), *as witness or victim* for “I would use it both as witness or victim” has a 30% rate (yellow). The most interesting result is that the majority of users, although they state to be satisfied with the application (*as witness* and *as witness or victim* reach a rate of 90%), would use the application just in case they act as witnesses of an accident: “In case I were directly involved into an accident I’d call the 112 number cause I’d been sure that they would come”. This is because they consider a traditional call a more direct way to communicate. In the next experiment we will discuss again these results.

The second part of the questionnaire is about each screen of the application and although the 60% of users considered the colors associated to the kinds of emergencies not useful or even confusing, all of them agreed about the other elements in the interface: icons, text and voice for the description, taking photos or videos, progress bar, and finally changing the geographical position.

### 4.2. Experiment in a Simulated Critical Situation

Here we introduce the second experiment that we performed with our application. It was executed in a real environment as simulation of an emergency scenario. Our goal with this experiment was to assess the benefits and drawbacks for the usage of our application in a real environment. Nevertheless, it is worth to remember that the EN application is not meant to replace traditional emergency phone calls, but to provide, on the one hand, citizens with an alternative channel and on the other, emergency organizations with richer notifications.

**Figure 3:**
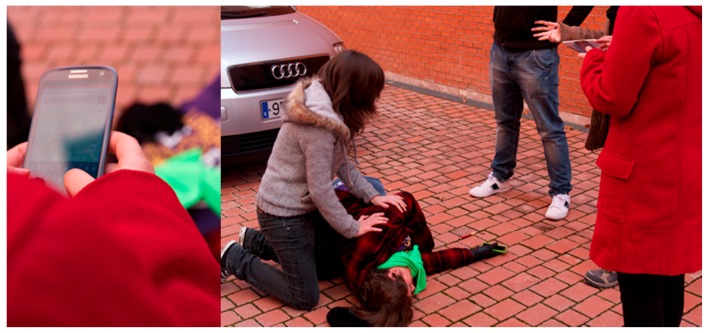
The simulated car accident in a university campus.

The scenario was selected during a focus group where researchers of our team and members of the Police Department of Valencia discussed the different possibilities. At the beginning we considered that the contributions in literature mostly consider large-scale crisis, as for example the Japanese tsunami in 2011 or Hurricane Sandy in 2012. Such scenarios refer to big crises occurred in USA and Asia where data coming from social networks were used to track and control the emergency evolution. However, during the focus group, it came to light that it is difficult to capture information about everyday emergencies through social networks. Actually an everyday scenario can give information on omitted aspects of EN apps that can be a real support for the common activities of an emergency organization. For this reason, together with the members of the Police Department of Valencia, we set up a simulated traffic accident in a street close to a university campus. It describes a victim laying down the street besides a car and a group of people acting as witnesses. We asked to some students to participate as actors simulating the victim and the witnesses. Figure 3 shows some scenes of the simulation.

We involved 10 potential stakeholders belonging to a different group of university students and researchers respect to the first study. Each session took around 15 min, in addition to the time spent interacting with the application and responding to a pre- and post-questionnaire.

Our testers were informed about the goal of the evaluation but not about the process. They just knew that they had to perform some kind of tasks with the mobile application. This choice is because we wanted to simulate the stress that a person could feel during an emergency situation. Indeed, when an emergency occurs, it generally takes people by surprise. In this way, we can test how easy it would be for users to understand and interact with this application in an uncommon situation.

The participants were requested to install the application on their phones and to play and interact with it a while before the experiment. In this way, they are already familiars with the mobile device. Before the installation they have been provided with a short documentation simulating the current practice of the mobile markets that provide users with some basic information and screenshots about the app to download.

Once the application was correctly installed, users were individually conducted to the emergency site. During the short walk they have been distracted and asked to speak about their life activities. This was to make users relaxed before the simulation starting and surprise them at the moment in which the simulation would have been executed. Indeed, when the users almost got their destination, the actors suddenly started simulating the accident with blood, shouts and noises. All the users started running to get quickly the place of the emergency and at this moment one of the actors asked them to alert the authorities. Considering that the users knew they were experimenting with a new notification application, they used it instead of calling an emergency number. Anyway, the simulation was executed so realistically that a pedestrian who was walking close to the simulation site tried to call the authorities. Moreover, there were several supervisors observing the experiment and taking notes of the users’ behaviors in a non-intrusive way.

#### **Questionnaire** 

Immediately after using the application, the participants were distanced from the simulation site and invited to answer the same questionnaire used in the usability test. In this way we had the possibility to better compare obtained results. This part of the experiment took approximately 15 min. 

**Figure 4:**
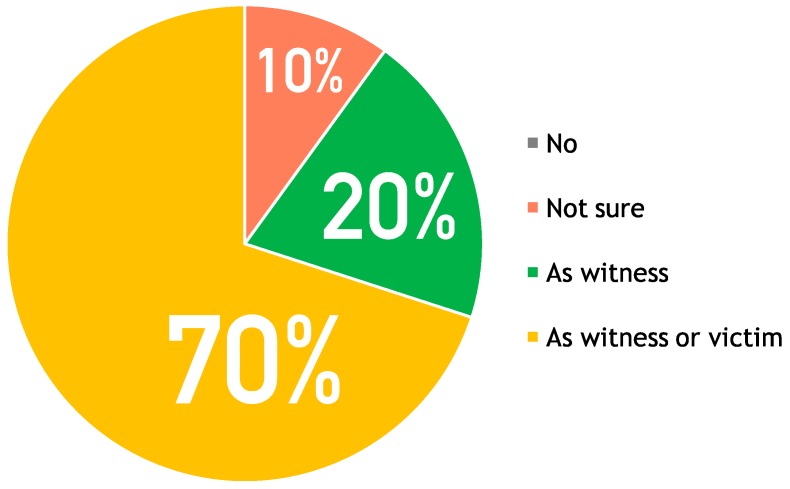
Overall user acceptance (would you use it?) of the proposed mobile application after the simulation.

Focusing on the acceptance, the users demonstrated to have been satisfied using the application. As shown in Figure 4, the majority of the testers would use the application as witnesses of an emergency or to call for help directly for themselves. This result is quite different from the usability test (see Figure 2), where a higher number of users would use it to report their testimony as witnesses of an emergency. This suggests that using the application in a real scenario would improve the utility perceived by the users. Nevertheless, there is still a small part of the users (20% rate, green in Figure 4) that consider the emergency call faster in case they are directly involved as victims and need help: “*In case I’d needed of immediate help I think a traditional call could be quicker*”.

To collect more detailed suggestions and comments, we have also interviewed the users face-to-face after the questionnaire. Some of our testers appreciated the experience with the mobile application. Three of them stated that shyness is often a big barrier and prevent them to make a voice call.

We have also collected opinions like: “*Sometimes I feel nervous and even shy to speak to the phone because strangers can listen to my conversation and I consider this a problem for my privacy*” or “*One day I was witness of a fire near my city and I didn’t contact the authorities cause I thought to be not able to describe what was happening*”. For them the application has the advantage to overcome this barrier: “*The mobile application guided me in describing the emergency and I feel comfortable using it more than calling an operator*”*.* Actually the mobile application presents just few and specific questions without requiring too much details. As shown in Table 3, our testers wrote just few words or even left blank the text area preferring to take pictures. In general, they considered the multimedia features (*i.e.*, GPS position, audio record, photos and/or video) quicker than writing and more conformable than making a call.

#### **Analyzing Data** 

We performed two kinds of analysis on the data retrieved during the experiment. The first analysis is aimed at interpreting the behavior of the users in terms of interaction. The second analysis, which has been performed as a walkthrough [33] with two emergency experts, is aimed to evaluate data on a quality level.

Table 3 resumes the data users sent to the system during the simulation. The mean time used to perform our experiment is 1:28 s, which can be compared with the average time coming from real data of similar accidents registered in the operation center of the Valencia Police Department that is 1:30 s. Even if the times used to inform the emergency agents are essentially the same, the benefits coming from using the mobile application are evident. In case of simultaneous calls, the emergency center needs one phone operator for each caller or the callers have to wait in order to be served. Using our system there is no need to wait to send a complete alert to the emergency organization. Moreover, the emergency experts recognized that in this way it is not needed to dedicate one operator to the analysis of each alert but the information can be automatically aggregated and monitored. However, this part mainly depends on the EM systems used by the operation center. During our collaboration with the police department of Valencia, we had the possibility to take advantage of an advanced information system currently employed for managing incoming emergency calls and plan how to easily adapt it for collecting messages from the mobile application.

Furthermore, Table 3 illustrates the data collected from the experiment in terms of words, multimedia files, time and user’s mistakes. From the table we can deduce that users prefer mainly taking pictures. Although they consider text and audio descriptions important, on average users wrote less than five words and just one recorded a short audio. Text and audio have been exploited just to underline the kind of accident or the presence of a victim. On average, each user took more than one photo, just one recorded a video. The average number of mistakes is irrelevant: 0.3. Errors are related to some contrasting information introduced by users, such as the wrong choice of the emergency category.

Differently from the lab experiment, in which users were requested to write a specific text, grab audio *etc.*, in this case they were free to interact as they preferred with the application. Consequently, they avoided to perform annoying and hard tasks as writing long texts and exploited the multimedia functionalities of the application. This increased the user experience even if the users were not in a comfortable situation such as an emergency simulation.

During the second analysis, the data were evaluated through a focus group involving two experts. The first is the chief of the operations room of the Police Department of Valencia. The second is the head of a company who develops EM Information Systems for the operation room. Both of them have collaborated in European and National projects for improving the management of critical situations. 

As summarized in Table 4, both experts highlighted some aspects linked to the kind of information the citizen can send. For instance, users are required to select in the application the most appropriate emergency type that in their opinion was unnecessary since it can be inferred by any EM operator looking at the description of the event. A second factor is about the choice of the users to send photos or videos rather than texts or audio recordings. Analyzing collected data from the simulation, operators can retrieve information much more detailed than the ones coming from traditional means. For example, observing the photos taken by the users the chief of the operation room noticed that the number plate of the car involved in the accident was clearly visible, recognizing that “*This is critical information often omitted by the citizens calling for help*”.

Another important consideration given by the experts is that emergency operators are able to recognize physical symptoms directly by observing a picture of the victim. Therefore, notifications based only on videos and photos taken by witnesses can be considered enough to create a new record in the emergency database. Moreover, the experts consider that textual and audio descriptions are not as relevant as the information coming from the photos. The texts reported in Table 3 are similar in terms of words and content and do not add information to the taken photos. A photo or a video are more objective and complete than a text elaborated by a person with limited or no experience in managing emergencies. Moreover, they highlighted the importance of multimedia with an example; few days before the interview, a flood occurred in the south of Spain. They commented: “*Do you know what was the most visited and broadcasted piece of news? It was one video recorded by a citizen with her smartphone*”. This underlines the crucial role that citizens have as witnesses situated in the first line when they have the right instruments to avoid understanding problems due to the lack of technical background.

The last comments were about the geographical location. Using the application, it is possible to gather both the emergency and the user location in an accurate way. This is evident especially if these data are compared with the information retrieved by the traditional emergency calls that rely on the capability of the person to describe her location. Indeed, experts stated that “*citizens often cannot give accurate information about their position and they just give the name of a nearby shop or describe the surrounding environment*”. 

## 5. Discussion

The experiments and the experience gained in designing and analyzing EN applications led us to point out some interesting findings. The first three considerations are about the usefulness, the acceptance by people and the usability of the application. First of all, EN applications can positively impact the current mechanisms used for notifying any exceptional circumstances. Indeed, even if the mean time to send a message through the application is slightly less than an emergency call, the real usefulness stands in the possibility for an emergency center to manage more than a notification at the same time with just one operator. Furthermore, the content of the notification is more accurate and complete than a traditional call.

Secondly, the application has been demonstrated to be accepted by potential users. The large majority of the interviewed users after the experiments declared that they would use this application when an emergency occurs. While in the lab study participants were more prone to use it as witnesses of an event, during the simulation they were surer to use it even as victims. This is due to the good experience they had during the simulation. Nevertheless, the EN applications are not meant to replace traditional phone calls, but only to provide citizens with a further official alternative channel.

Regarding the usability, the experiment demonstrated that the application is *easy-to-use* and the interviews show the level of the possible acceptance is good. Finally, also the observation of the users’ behaviors gave us interesting findings. Our users turned out to be expert in gathering information; they did not show any hesitation in managing the phone and catching data. As a matter of fact, people are already familiar with all the main functionalities integrated in our application and use them confidently. This is paramount since people in the real world will use an emergency system just when they are already used to it [34].

Another point to discuss is the different usage of mobile EN applications and traditional social software when an emergency occurs. Apparently, their scope does not completely overlay. Social networks are normally exploited by emergency organizations to catch information about big-scale crises, as for example a tornado hitting a large region. In other words, the organizations know about the emergency and they look for fresh information coming from people directly on the disaster site. Otherwise, content related to small-scale emergencies would not emerge from the great amount of data generated by social media. For this reason, dedicated EN applications are suitable to keep organizations informed especially about small-scale crises, in particular if authorities are not aware about them.

Finally, we also found out some useful technical aspects to take into account for designing EN applications. The important factor for these applications is clearly the *multimediality*. Indeed, we observed that photos combined with the automatically retrieved position are the main sources for the messages. Users tend to write just short texts or even nothing. The text can be used as complementary medium or an alternative when specific conditions do not allow to use other media (e.g., low light for photos/video, noise for audio grabbing *etc.*).

The accuracy level of the embedded GPS is considered adequate both by the experts and users (*i.e.*, *localization*). This avoids further interactions as using a map or writing a formal street address to localize the position. Concerning the minimum information needed to create an emergency alert, the user position combined with the user profile (e.g., phone number and full name) is enough as stated by interviewed emergency experts. Storing the personal information and retrieving automatically the device position would allow to send an emergency notification just in one step.

The last findings are related to the *application interface*. Users are generally annoyed by long tasks and do not pay enough attention, much less during emergency situations. So that designers should elaborate a direct and short navigation through the application otherwise users will prefer traditional calls. Also the language is important because users can be confused and upset by displayed instructions. For this reason, short, easy and understandable labels and descriptions are needed. Finally, *feedback* is a critical factor for an emergency application. Indeed, during a traditional call users are reassured by the voice of an operator. The mobile application must be able to provide a similar response with, for example, a confirmation message about the successful delivery of the notification and the decisions made by the operators.

## 6. Redesign

**Figure 5:**
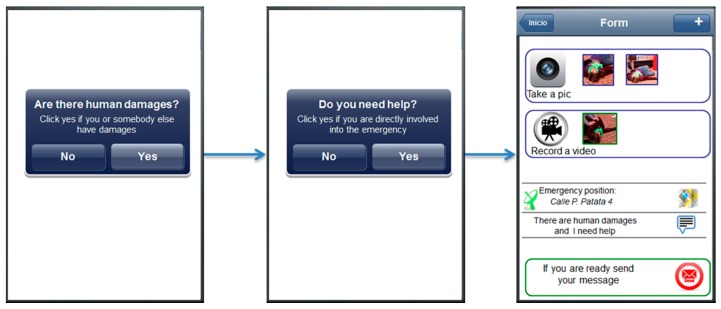
The mobile application after the re-design process.

On the basis of the findings pointed out in the previous section, here we propose a possible redesign of the application. About the *application interface*, long forms could annoy and frustrate the users and for this reason the total number of steps in the application has been significantly reduced and the interface simplified, as shown in Figure 5. Following with this idea, the category view has been eliminated considering that the selection of the right category is an expert task and can lead users into error. Also labels and messages have been changed to be more familiar and understandable to users. About *multimediality*, the multimedia section has been moved in the top of the application, because even if its usage is optional, the position and the graphic aspect improve its emphasis.

Related to the findings about *application interface* and *feedback*, some automation features have been introduced to help users in elaborating the alert:

- A textual description is pre-filled thanks to the questions placed at the very beginning (see the first two screens in Figure 5). If needed, users can modify it.
- User profile is preregistered into the system while her position is automatically retrieved by the application. In case, the users can just send the message without adding other information.
- The users’ position is retrieved on the go and displayed to the users that can modify on a map.
- Photos previously taken in a certain period of time (e.g., few minutes before) are preloaded into the application considering that they are probably related with the particular circumstance and the users would add them to the notification.
- The quality of the mobile network is evaluated and, in case it is not good, users are recommended to avoid multimedia data and insert just a textual message. Finally, the application shows an alert to the users when their message is received by the system and when an operator endorses an emergency operation for them.

## 7. Conclusions

In this paper, we investigated EN mobile applications with the aim to analyze the main characteristics and understand their effective usefulness in the real world. We carried the study on using the DSR approach by Hevner and Chatterjee [8]. Following this practical approach, we designed a new artifact with the goal to learn.

Therefore, in our research process we started considering first of all the literature in the context of the social applications applied in the EN area, and then the relevant specific EN applications currently available. From this analysis we found that generic social applications are generally used to collect information about large-scale crises while specific applications are effective also in small-scale events. Moreover, the majority of the EN applications share common characteristics and some limitations. Indeed, the message content is often based on text, forms, GPS position and photos. The emphasis is given especially to text and forms. Focusing on their design, we noticed that their usability is not well treated and considered. The result is that users are often required to fill out complex forms with too much and useless information. Moreover, the message content is mainly based on text that can misled the user and make the task completion slower.

Taking into consideration these results, we developed a new mobile application for notifying emergencies. In particular, we tried to overcome some of the usability limits identified in the state of the art. To have a feedback about the proposed design, we evaluated the application performing two different experiments with both citizens and emergency practitioners.

On the basis of these evaluations and the experience gained along the entire research process, we have pointed out some findings related to both the usefulness of such applications and the possibility of their effective real adoption. The most crucial ones are about the interaction of users with these tools during critical situations. Our conclusion is that people are already familiar with the mobile technology and they are keen on using it also for notifying emergencies. Moreover, while the EN applications work mainly with text and forms, from the experiment it is clear that users, when they can, avoid these resources and prefer the multimedia aspects that on the other side looked to be more complete and precise from the point of view of the emergency agents.

Finally, the whole design process and the corresponding findings can serve as a reference point for further research in the same direction. In particular, our contribution wants to be a support for those practitioners who try to design EN applications without enough experience or resources for testing their solutions and improving both efficiency and effectiveness.

As future works, we plan to effectively integrate the application with the emergency information system currently used by the Police Department of Valencia (Spain). In this way, we aim at reaching a wider users base and extracting more quantitative and qualitative data for evaluating the proposed application. Moreover, we want to move our focus on other phases of the emergency management process, defined as preparedness, recovery and mitigation in [35], in order to study the applicability of mobile technologies. Also in this case, first of all it would be crucial to clarify the role of citizens within these phases and how their participation can be helpful for the operation center.

## Figures and Tables

**Table 1:** Analyzed mobile applications with their main features.

EN App	Data Shared with Citizens	Recipients	Media	Text	GPS	Call	Other Functions	Interface Style
**SafetyGPS** Version: n/r; O.S.: Android	Twitter	List of city halls	•	•	•			Console interface
**HelpBridge** Version: 2; O.S.: Windows	Social network Georeferenced multimedia message Phone line	Contact list of friends and family	•	•	•	•	Donations Volunteers	Simple textual form
**Motorola Alert** Version: 1.02.53; O.S.: Android	Georeferenced message	Contact list of friends and family			•	•	Sound alert Meet me	Simple interface based on a map widget
**EmergencyAlert** Version: 1.0.2; O.S.: Android, iOS	Georeferenced message	Contact list of friends and family		•	•			Long and complex forms
**FEMA App:** Version: 2.6.5; O.S.: Android, iOS, BlackBerry	Shared map	FEMA Citizens	•	•	•		Safety tips Emergency kits	Long and complex forms
**SignAlert** Version: 3; O.S.: Android, iOS	Social network	Citizens	•	•	•			Long and complex forms
**ELERTS** Version: 2.2; O.S.: Android, iOS	Georeferenced multimedia message	Security department	•	•	•	•		Simple textual form combined with a map widget
**My112 & similar** Version: 1.0.8; O.S.: Android, iOS	Georeferenced message	112 center			•	•	Receiving alerts	Simple interface based on a map widget

**Table 2:** Subtasks of the usability study.

Task ID	Task Description	Untrained Mean Time (min)	Trained Time (min)	Untrained Mean Errors (number)	Trained Errors (number)
1.1	Select the category of the emergency	0:15	0:09	0	0
1.2	Take 2 photos of the accident (the targets are assigned before the task)	0:30	0:28	0	0
1.3	Describe the accident (a text is provided during the experiment; 160 chars—the length of a sms)	1:39	1:32	0.2	0
1.4	Find the accident position	0:25	0:18	0	0

**Table 3:** Quantitative data resulting from the experiment.

User	Words	Text	Audio	Video	Photo	Localization	Time (Minutes)	Errors
01	7	Traffic accident, person wounded in the head	-	-	1	Automatically retrieved with GPS	00:50	0
02	11	Unconscious man with brain trauma. no documentation of the injured person.	-	-	2	Automatically retrieved with GPS	01:30	0
03	5	Accident near University Carlos III	-	-	1	Automatically retrieved with GPS	01:20	0
04	11	Hit man in the ground, injured head, unconscious and unresponsive state	-	-	2	Automatically retrieved with GPS	02:13	0
05	1	Wounded	-	-	2	Automatically retrieved with GPS	01:17	0
06	-		-	-	1	Automatically retrieved with GPS	02:01	1
07	1	Accident	-	-	1	Automatically retrieved with GPS	01:30	1
08	-		7 s	2 s	2	Automatically retrieved with GPS	01:00	0
09	3	Road accident, wounded	-	-	1	Automatically retrieved with GPS	01:50	0
10	-		-	-	1	Automatically retrieved with GPS	01:10	1

**Table 4:** Summary of experts’ considerations.

Citizens should not have been required to classify the kind of emergency because they do not have the adequate competences and because EM operators can do it without particular efforts thanks to the information received from the mobile system. Videos and photos taken by users are considered enough to create a new record in the emergency database. The textual and audio descriptions provided by users are short and not as relevant as the information coming from the other media. The information retrieved by the mobile application about the emergency and user positions is much more accurate than the one EM operators can retrieve by speaking directly with a citizen.
